# Characteristics of iodine-bearing silver-impregnated alumina sorbents and their direct solidification *via* hot isostatic pressing

**DOI:** 10.3389/fchem.2023.1089501

**Published:** 2023-01-23

**Authors:** Tomofumi Sakuragi, Satoshi Yoshida, Osamu Kato

**Affiliations:** ^1^ Radioactive Waste Management Funding and Research Center, Tokyo, Japan; ^2^ Kobe Steel Ltd., Kobe, Japan

**Keywords:** waste management, TRU waste, iodine, AGA, sorption, mass transfer zone, immobilization, hot isostatic press

## Abstract

Waste management for radioiodine is a key issue for the sustainable nuclear fuel cycle. The iodine adsorption behavior on a bed column of a silver-impregnated alumina sorbent (AgA) under conditions designed to match those of the Rokkasho reprocessing facility dissolver off-gas (DOG) system was investigated using different volatilized iodine concentrations. Cross-sectional observations of iodine-bearing AgA grains revealed that iodine was adsorbed as silver iodide and silver iodate, and gradually distributed from the surface to the inside of the AgA. The iodine distribution throughout the AgA beds allowed us to estimate the length of the mass-transfer zone. This suggests that the iodine load fraction in AgA (adsorbed iodine/total impregnated silver) will be averaged to 50% in the expected facility equipment design. This study also describes the waste form durability after disposal. To reproduce the average iodine loading in the waste form, 100%-loaded AgA grains were mixed with an equal amount of commercially available alumina reagents and consolidated through hot isostatic pressing at 175 MPa and 1,325°C for 3 h. The resultant 50%-loaded solid was used for the static leaching test over 4.5 years, where the leached iodine was less than 0.2% under simple reducing conditions. This suggested that the HIPed solid of AgA from Rokkasho DOG showed preferable water resistance for after disposal safety.

## 1 Introduction

Silver-impregnated materials are widely used as radioactive iodine adsorbents in the nuclear industry. The nuclear fuel reprocessing facility in Rokkasho, Japan contains several off-gas treatment systems, including the dissolver off-gas (DOG), vessel off-gas, and melter off-gas, which remove radioiodine. The DOG stream has the highest iodine concentration, where the radioiodine discharged from spent fuel dissolution is removed by the silver-impregnated alumina sorbent (AgA) to prevent radioactive emission to the environment. The total amount of spent AgA and total ^129^I inventory are estimated to be 137 tons and 5.1 × 10^13^ Bq, respectively, from 40 years of reprocess operation at 32,000 MTU (Federation of Electric Power Companies (FEPC) and Japan Atomic Energy Agency (JAEA), 2007); The Nuclear Waste Management Organization of Japan (NUMO, 2018). Waste management of spent AgA for disposal deep underground is a key issue, because ^129^I has an extremely long half-life (1.6 × 10^7^ years) and is mobile in engineered and geologic barrier systems. Thus, additional stabilization treatments for iodine-bearing waste are needed to meet the long-term performance requirements of waste disposal.

Since the 1980s, several waste forms, such as simple iodine compounds, glasses, and iodine-bearing minerals, have been suggested for iodine immobilization (Audubert et al., 1997; Vance and Hartman, 1999; Hyatt et al., 2003; Sakuragi et al., 2008; Stennett et al., 2012; Haruguchi et al., 2013; Jubin and Bruffey, 2014) and were reviewed by Riley et al. (2016). In recent times, iodine immobilization remains a significant concern for reprocessing and subsequent waste management, thus warranting further dedicated research on durable waste form development (Idemitsu and Sakuragi, 2015; Mukunoki et al., 2016; Sakuragi et al., 2016; Yamashita et al., 2016; Coulon et al., 2017; Yang et al., 2017; Vance et al., 2018; Asmussen et al., 2019).

Hot isostatic pressing (HIP) is a promising consolidation treatment for iodine immobilization to produce durable iodine-bearing waste (Fujihara et al., 1999; Vance et al., 2005; Sakuragi et al., 2015; Masuda et al., 2016; Matyas et al., 2016; Maddrell et al., 2019). Direct consolidation of spent AgA is advantageous, as it is a simple process that does not generate secondary waste. This is because alumina, a base material of AgA, can be used as a solidification matrix without iodine desorption from the spent AgA. The waste form characteristics are thus characterized by the properties of spent AgA, such as iodine loading. However, little to no information was found on the actual iodine load fraction (*f*_*load*_) relevant to the DOG system of the Rokkasho reprocessing facility. In previous studies, several simulated waste forms were synthesized using AgA with an iodine loading of 100% (Sakuragi et al., 2015; Masuda et al., 2016).

Gas-phase iodine capture has been studied in silver sorbents using inorganic or organic iodine (Fukasawa et al., 1994; Sakurai et al., 1997; Takeshita and Azegami, 2004; Maddrell et al., 2015; Yang et al., 2015; Bruffey et al., 2016). In every case, the adsorption bed length was as small as 100 mm or shorter (Fukasawa et al., 1994; Sakurai et al., 1997; Takeshita and Azegami, 2004; Bruffey et al., 2016), or batch adsorption was performed (Maddrell et al., 2015; Yang et al., 2015). The iodine filter columns of the Rokkasho DOG have a bed length of 850 mm. This study investigates the adsorption of stable iodine onto AgA in a scaled-up system under simulated conditions of the Rokkasho DOG stream. Iodine filters are replaced when a breakthrough occurs. Thus, the mass transfer zone (MTZ) of iodine in the bed column provides information on the *f*_*load*_ of spent AgA in the DOG. Based on the MTZ results, the targeted load fraction of AgA was reproduced by blending the saturated AgA (100% load) with alumina powder; then, the simulated AgA was consolidated using HIP. A static leach test over 4.5 years was performed under reducing conditions using the HIPed solid.

## 2 Experiment

### 2.1 Iodine adsorption experiment

Silver-impregnated iodine sorbents (AgA) used in the DOG treatment system of the Rokkasho reprocessing facility were obtained. The silver content of the porous AgA beads was over 10% by weight in nitrate form. AgA specifications are listed in Table 1.

**TABLE 1:** Specification of silver-impregnated iodine sorbents (AgA).

Silver content	≥10 wt%
Packing density	500–1,000 g/L
Color	White
Silver speciation	AgNO_3_
Base material	Alumina (Al_2_O_3_)
Spherical grain size	Approximately 1.7 mm

Adsorption experiments were performed by passing iodine through the AgA beds. As a preliminary test, small-scale adsorption experiments were performed using three sorbent beds placed in series. The first, second, and third beds were 20 mm deep, had a 54 mm inner diameter, and were separated by 25 mm thick glass wool in the same glass column. Iodine (I_2_) at 160 ppm was passed through the column beds maintained at 150°C for 6 h. The load fraction, speciation, and distribution of the iodine adsorbed on the sorbents were investigated.

**FIGURE 1:**
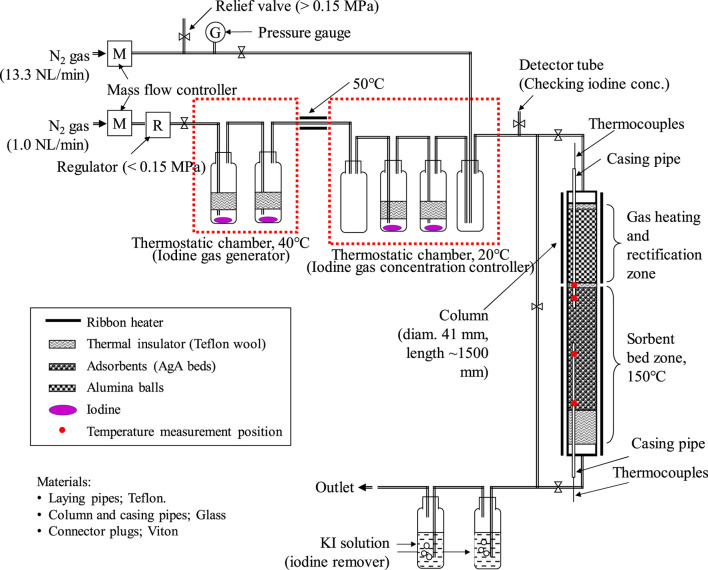
Schematic diagram of iodine adsorption onto AgA beds in the column experiment.

In the next step, adsorption tests were performed using the column system under low and high iodine concentrations and different bed lengths up to 1,200 mm. The scale-up tests using the actual adsorbents better simulated the Rokkasho DOG system in terms of bed length, temperature, and flow rate. Iodine concentrations are assumed as high and low, because the concentration cannot be adjusted exactly to that in the DOG. The HNO_3_ concentration in the off-gas was not considered in this study because they are recovered before iodine adsorption. Figure 1 shows the apparatus used for column adsorption tests. The carrier nitrogen gas flowed through the system. Iodine was generated in the first thermostatic chamber, composed of two containers containing iodine maintained at 40°C. The iodine concentration was controlled in a second thermostatic chamber kept at 20°C, where iodine was diluted using nitrogen gas supplied from another line. The iodine was flushed through a bypass line until the concentration remained constant. Then, a given concentration of iodine was pre-heated and rectified in the inlet of a column filled with alumina balls and passed through the adsorption bed filled with AgA beads at 150°C. The temperature inside the bed was monitored during the test. The operation times were set taking the iodine concentration into consideration. The column test conditions are summarized in Table 2.

**TABLE 2:** Conditions of iodine adsorption onto AgA beds in the column experiment.

Iodine concentration	8 ppm		160 ppm	
Carrier gas	N_2_			
Column size	Inner diameter 41 mm, length ∼1,500 mm			
Bed length	100 m	900 mm	100 mm	1,200 mm
AgA weight	150 g	1,300 g	150 g	1750 g
Gas flow rate	22.2 L/min (14.3 normal L/min)			
Liner velocity	0.28 m/s			
Temperature	150°C			
Operation time	120 h	360 h	6 h	360 h

After the adsorption tests, AgA was collected from a predetermined location in the beds. The elemental distribution in the AgA beads was observed using an electron probe micro-analyzer (EPMA) (JEOL, EPMA8800RL). After crushing and dissolving, the amounts of Ag and I loaded on AgA were measured using inductively coupled plasma atomic emission spectroscopy (Shimadzu, ICPS-800), and NO_3_^−^ and IO_3_^−^ were measured using ion chromatography (DIONES, DX-500, ICS-3000).

### 2.2 Static leaching test of HIPed solid

#### 2.2.1 Consolidation of AgA by HIP

Based on the results obtained from the iodine adsorption experiments, the simulated 50% iodine-loaded AgA was consolidated using HIP. In the present work, 50%-loaded AgA simulants were prepared by blending α-alumina with saturated AgA (100% iodine loading), obtained by loading non-radioactive iodine onto virgin AgA and passing it through excess I_2_ at 150°C. The 100%-loaded AgA was crushed into particles of approximately 40 μm before mixing. The α-alumina (Taimicron, TM-DAR) was obtained from Taimei Chemicals Co., Ltd. This alumina powder has a high purity over 99.99%, BET surface area of 14.5 m^2^/g, diameter of 0.10 μm, and sintered density of 3.96 g/cm^3^. Two materials were uniformly mixed in an equivalent weight ratio and the mixture was encapsulated in a stainless steel can (50 cm^3^) and heated at 450°C under high vacuum (<133 × 10^−5^ Pa) for 2 h to remove volatile components. This thermal treatment before HIP allows all iodine species to change to silver iodide (AgI) and the solid to become denser (Masuda et al., 2016). The HIP treatment was then performed at 1,325°C and 175 MPa for 3 h using an Ar gas-pressurized HIP system (Kobelco, Dr. HIP). The solid porosity was determined using the Archimedes method (Japanese Industrial Standards, 1992).

#### 2.2.2 Leaching test procedure

The HIPed solid with 50% iodine loading for the leaching test was cut into 10 mm cubes and washed with a dilute sodium sulfide (Na_2_S) solution (3 × 10^−3^ M) under ultrasound for 20 min to remove the AgI exposed on the specimen surface. This procedure was repeated five times, and the samples were rinsed with deionized water. Less than 1% of the total iodine in the solid was lost during the washing procedure. The static leaching test was carried out in 600 ml deionized water (surface-to-volume ratio of 0.01 cm^−1^) for 1,687 days at room temperature (22°C–23°C). The immersed sample was kept in a glove box purged with N_2_ gas (oxygen concentration <1 ppm). During the test, the pH and Eh of the solution were checked regularly. A small portion of the 600 ml leaching solution was obtained for each sampling period to analyze the concentrations of the leached elements using ICP-MS (PerkinElmer, ELAN DRC II and Agilent 7,700x). After 1,687 days, the solid was removed, and the cross-section was observed using the EPMA.

## 3 Results and discussion

### 3.1 Iodine adsorption experiment

**FIGURE 2:**
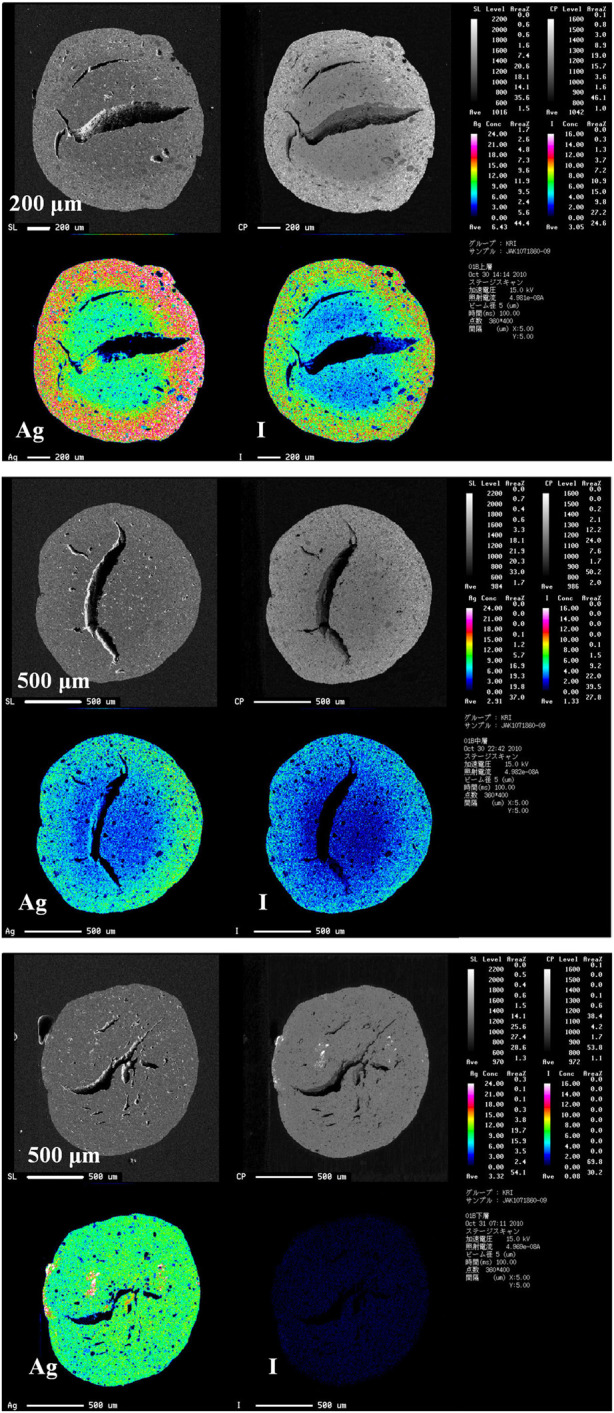
Electron probe micro-analyzer (EPMA) images for AgA cross-section AgA after iodine adsorption in a small-scale adsorption experiment equipped with three bed columns. The top, middle, and bottom images represent iodine-bearing AgA obtained from the first, second, and third beds, respectively. The iodine loadings are summarized in Table 3.

Figure 2 shows the EPMA cross-section images of AgA after iodine loading in the small-scale three-bed adsorption experiment. Large voids could be observed in the AgA. Iodine co-existed with silver and was distributed from the outside to the inside of the AgA, except for the third bed. This suggests that the silver was impregnated heterogeneously in the original AgA, and that the iodine adsorbed onto the silver to form compounds, such as AgI and AgIO_3_. The presence of AgI and AgIO_3_ was confirmed through X-ray diffraction (XRD) analysis of 100%-loaded AgA (Masuda et al., 2016).

The $f_{load}$ in AgA can be obtained from the following equation:

$$f_{load}=\frac{M_{iodine}}{M_{silver}}$$

where $M_{iodine}$ and $M_{silver}$ represent the moles of adsorbed iodine and silver impregnated on AgA (mol/g-AgA), respectively. The load fraction decreased from 0.77 to 0.22 in the order of the first, second, and third beds. The characteristics of iodine-loaded AgA in small-scale adsorption experiments are summarized in Table 3. The AgIO_3_ to AgI ratio in AgA was nearly constant at < ∼0.3, regardless of the $f_{load}$ .

**TABLE 3:** Iodine loading on AgA obtained from the small-scale adsorption experiment using three beds.

	AgNO_3_ (mmol/g)	AgI (mmol/g)	AgIO_3_ (mmol/g)	AgIO_3_/AgI	Iodine load fraction (*f*_*load*_)
First bed	0.348	0.900	0.240	0.27	0.77
Second bed	0.660	0.540	0.156	0.29	0.51
Third bed	0.906	0.199	0.054	0.27	0.22

**FIGURE 3:**
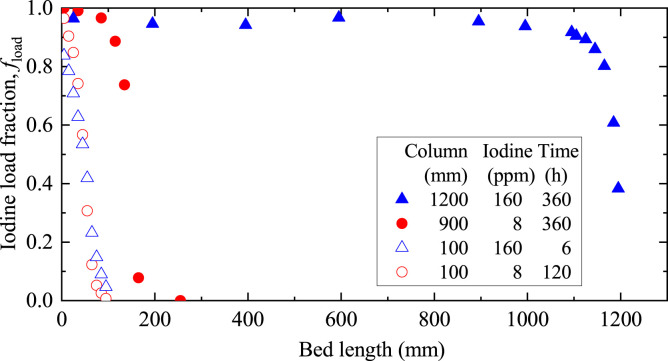
Iodine adsorption behavior onto AgA beds in the column experiments.

Figure 3 shows the iodine adsorption behavior onto the AgA bed column in the scale-up experiments to estimate the realistic load fraction of spent AgA in the DOG. The *f*_*load*_ decreased rapidly with bed length, but reached the outlet of the column in both short-column tests, suggesting a breakthrough from the 100 mm beds. Owing to the low iodine concentration of 8 ppm, the *f*_*load*_ values also decreased to zero at the inlet in a bed length of 300 mm for the 900 mm column. Thus, the MTZ (*f*_*load*_ = 0.05–0.95) was not clear for the 900 mm column. In the 1,200 mm column test with 160 ppm iodine shown in Figure 3, the fully loaded fraction decreased from 600 mm to 900 mm, and then *f*_*load*_ values decreased to 0.5 at 1,190 mm. Considering the symmetry and the midpoint of 750 mm between 600 mm and 900 mm as the *f*_*load*_ value of 0.95, the MTZ length could be estimated at 880 mm. The iodine filter columns of the Rokkasho DOG have a bed length of 850 mm and will be replaced when breakthrough occurs. Thus, the average *f*_*load*_ value of the spent AgA was roughly estimated as 50%. The difference between iodine concentrations of 8 and 160 ppm may have been too large. The MTZ data might not be sufficient for detailed verification; however, the present study assumed a 50% loading for subsequent investigations regarding AgA solidification and its leaching behavior.

### 3.2 Static leaching test of HIPed solid

**FIGURE 4:**
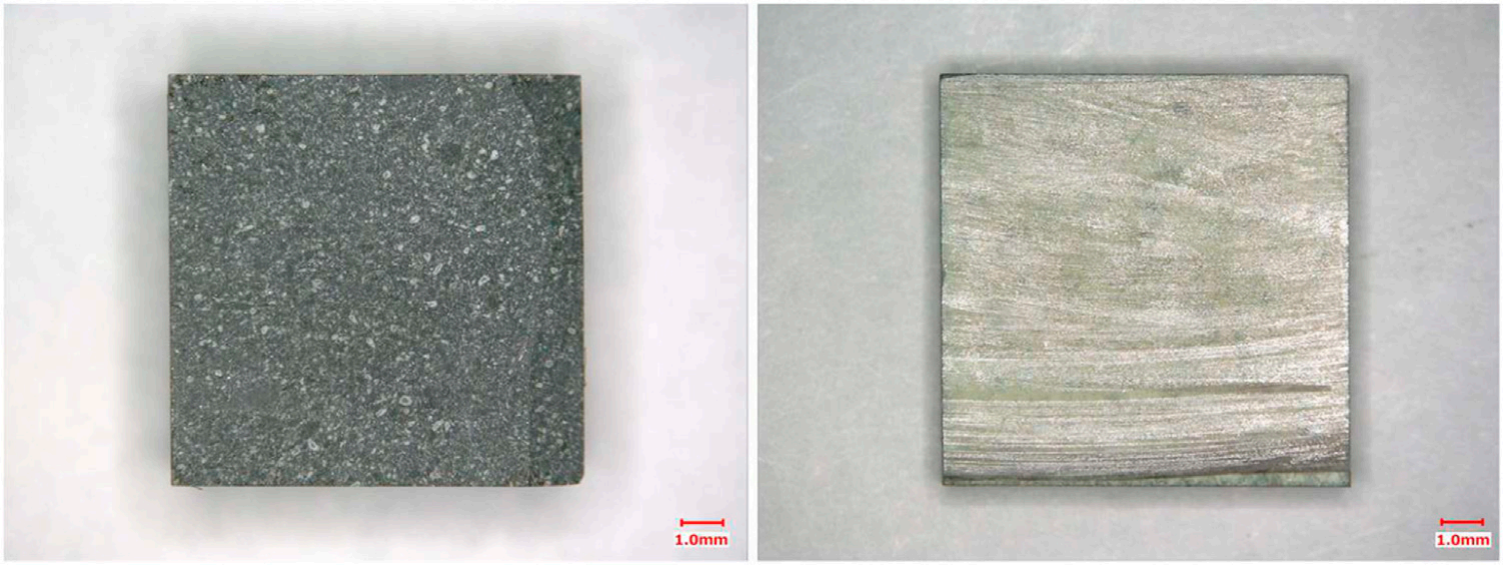
Photographs showing 50% solid loading after immersion test. The left represents the solid surface. The right represents the cross-section.

The porosity of the HIPed solid synthesized using the simulated 50% iodine-loaded AgA was 4.4%. This porosity corresponds to previous data for a 100%-loaded solid (Masuda et al., 2016). The HIP process for iodine-bearing AgA confirmed that fine AgI was retained uniformly in the crystal grain boundaries of the α-alumina (Al_2_O_3_) matrices (Sakuragi et al., 2015; Masuda et al., 2016). Figure 4 shows the appearance of 50% solid loading after immersion. The solid surface (left image) was dark gray, because the sample was washed with Na_2_S solution before immersion, suggesting that silver sulfide (Ag_2_S) formed on the surface as follows: 2AgI + HS^−^ = Ag_2_S + 2I^−^ + H^+^. Inagaki et al. (2007) used XRD to confirm the formation of Ag_2_S on the surface of AgI particles in contact with Na_2_S solution. Alteration was limited only to the surface and the light gray area in the cross-section represents pristine α-alumina and slight, fine AgI.

**FIGURE 5:**
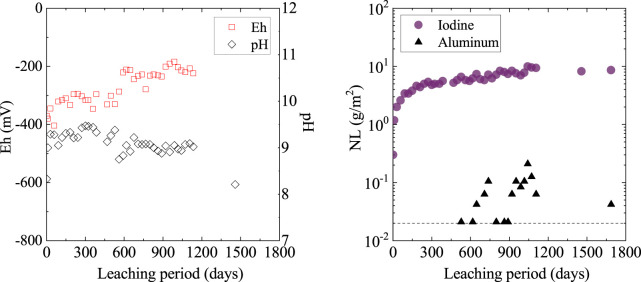
The normalized mass loss (NL) of iodine and aluminum during the static leaching test using 50% solid loading. The dashed line represents the aluminum detection limit.

The leaching test results are shown in Figure 5. We obtained the normalized elemental mass loss (*NL,* g/m^2^) using the following equation:

$${NL}_{i}=\frac{C_{i}}{f_{i}}\times \frac{V}{S}$$

where $C_{i}$ is the mass concentration of element i in the leachate (g/m^3^), $f_{i}$ is the mass fraction of element i in the specimen (i.e., 50% loading solid), V is the leachate volume (m^3^), and S is the geometric surface area of the specimen (m^2^). The initial neutral pH and reducing conditions were mostly maintained for up to 1,200 days. The Eh and pH data after 1,200 days were missing, but were expected to show no significant change, because the leaching data for I and Al remained constant after 1,200 days. The leached Al was retained around or below the detection limit. The iodine leach was larger than that of Al. The ${NL}_{I}$ values were larger than the *NL*_*Al*_ values by a factor greater than 10^2^. This incongruence between the solid matrix (alumina) and immobilized iodine dissolution is attributed to the difference in solubility. Alumina is known for its extremely low solubility in neutral pH conditions. Under reducing conditions, AgI is thermodynamically unstable and can be altered *via* the following reaction: AgI + e^–^ = A(s) + I^−^. During the leaching test, the Eh values were kept at approximately—400 to −200 mV. However, the ${NL}_{I}$ values in the present work that increased rapidly in the first stage showed a slight increase or were nearly constant after 300 days, with values around 10 corresponding to a leached ratio of less than 0.2%.

**FIGURE 6:**
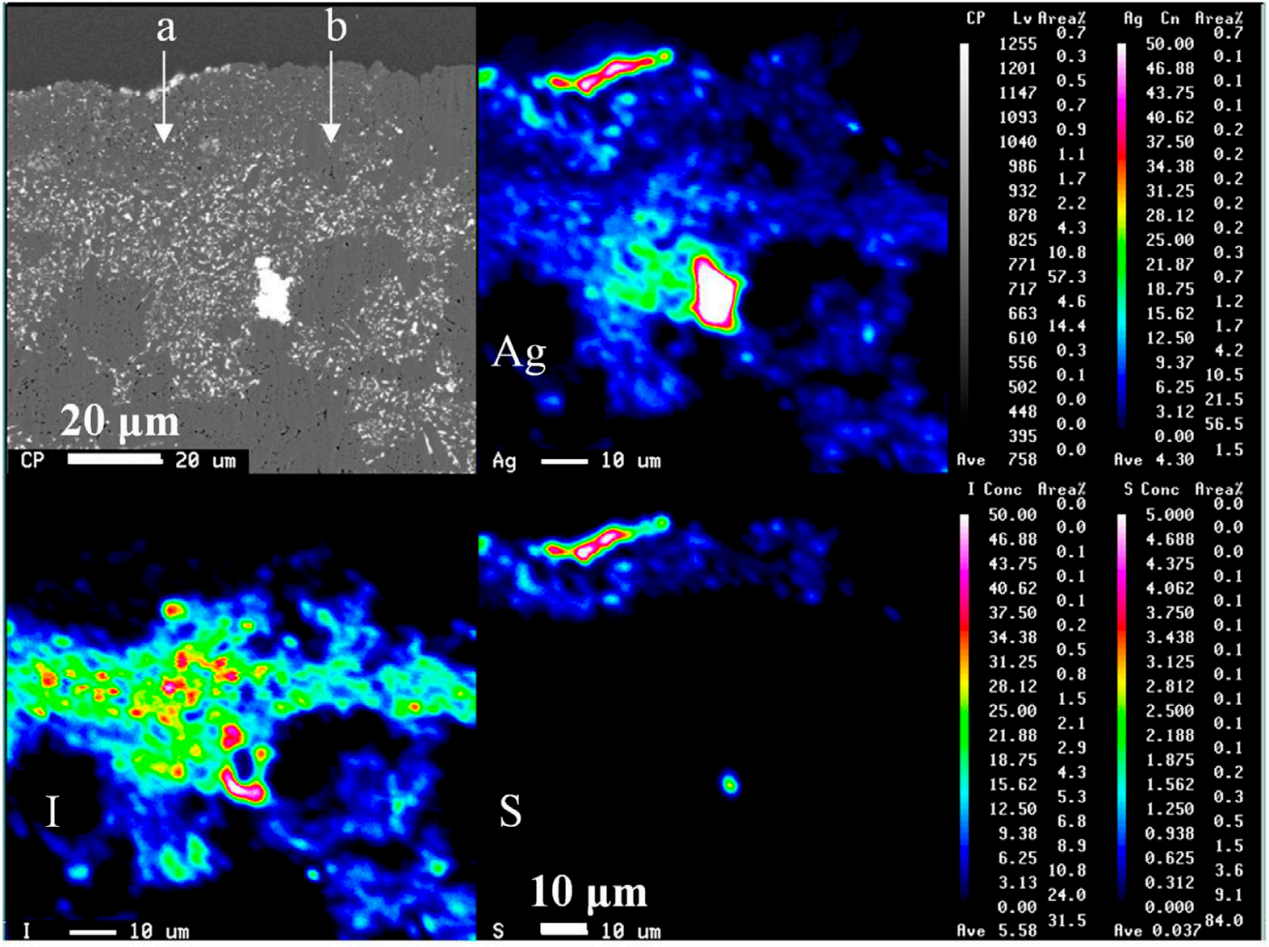
EPMA analysis of cross section at 50%-loaded solid after 1,687 days of aqueous immersion. The top of images shows the solid/water interface. The symbols a and b represent the direction of line analysis in Figure 7.

**FIGURE 7:**
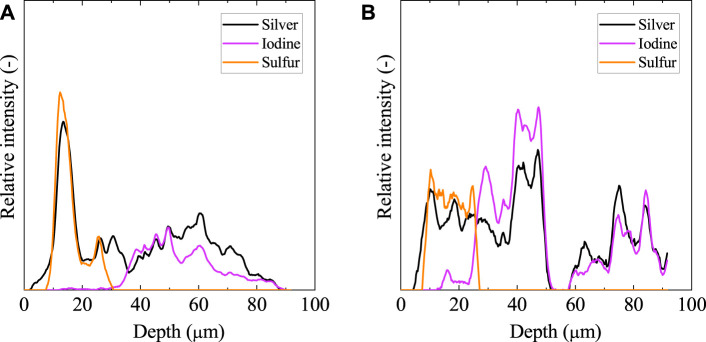
Elemental distributions from the surface to the inside for the 50%-loaded solid after immersion obtained in Figure 6. The left **(A)** and right **(B)** represent the results for the a and b directions in the EPMA image, respectively.

Figure 6 shows the elemental distribution of the solid cross-section after the leaching test using an EPMA. Figure 7 also shows the element distribution *via* line analysis from the surface to the inside of the solid, obtained from the EPMA results in Figure 6. Silver co-existed with sulfur and iodine at the solid surface and inside, respectively. Sulfur was located only on the solid surface as Ag_2_S due to surface washing before immersion, as mentioned previously. Iodine was distributed immediately below the surface of the Ag_2_S layer. Evidently, only a small amount of iodine was released from the solid.

The above results suggest that the dense 50%-loaded solid showed preferable water resistance under reducing conditions, unless aggressive reducing agents, such as hydrosulfide, were present. The kinetic study of AgI dissolution supports the present results, as iodine release proceeds at an extremely slow rate because of the formation of a protective Ag layer at the AgI surface under reducing conditions with Fe^2+^ (Inagaki et al., 2008). However, under high hydrosulfide conditions, iodine leaching from HIPed solids is accelerated by a strong silver-sulfur reaction (Sakuragi et al., 2015).

## 4 Conclusion

In the present work, realistic 50% iodine loading onto AgA was achieved *via* iodine adsorption experiments using a 1,200 mm length column under simulated conditions for the DOG system in the Rokkasho reprocessing facility. A simulated 50%-loaded solid was synthesized using HIP, and its durability and iodine immobilization performance were investigated for long-term disposal safety. Over 4.5 years, the leaching test under reducing aqueous conditions showed that leached iodine was less than 0.2% of the iodine in the solid owing to the protective role of the dense Al_2_O_3_ matrix and the passive layer on the AgI surface. These results suggested that the HIP solidification for iodine-bearing wastes from the nuclear fuel cycle is promising for the waste management to meet the long-term disposal safety. It will be a future challenge to investigate the detailed leaching process relevant to the microstructure of alumina matrices. The performance of the solid under more severe conditions, such as higher pH conditions, where the alumina matrix dissolves is of interest.

## Data Availability

The original contributions presented in the study are included in the article/Supplementary Material, further inquiries can be directed to the corresponding author.
